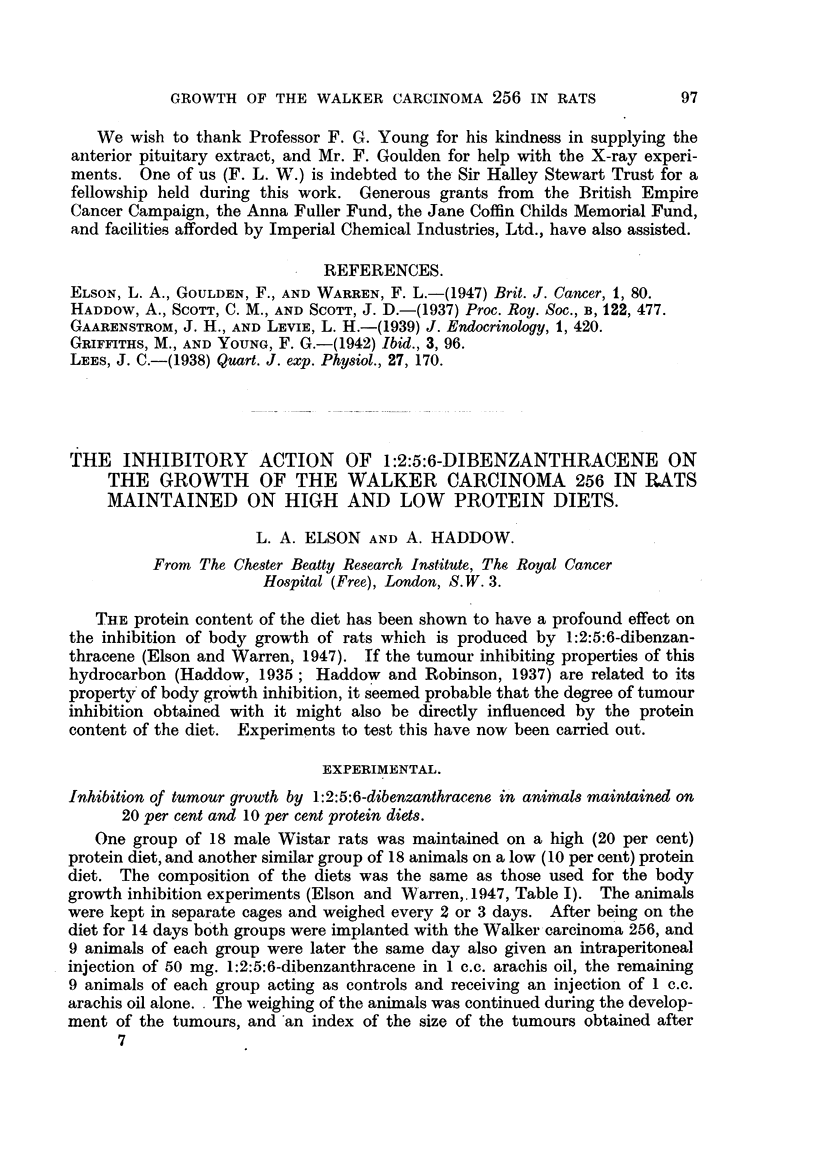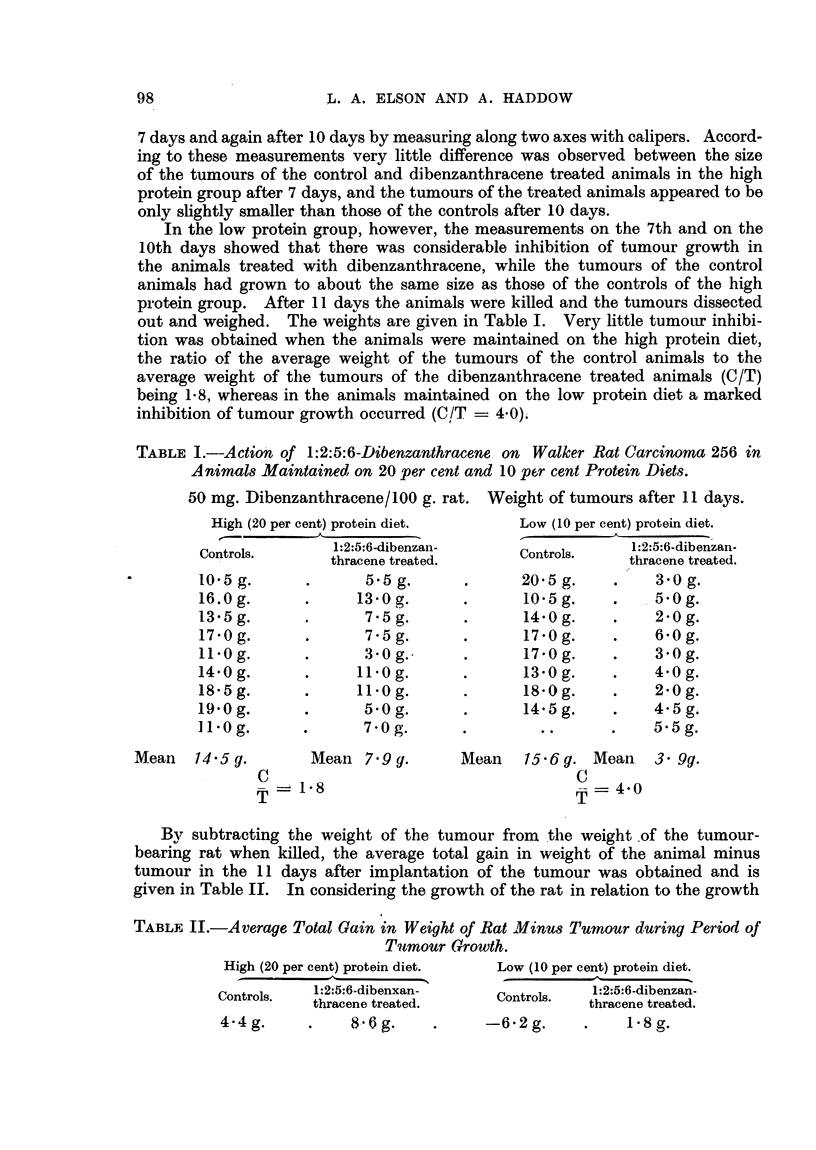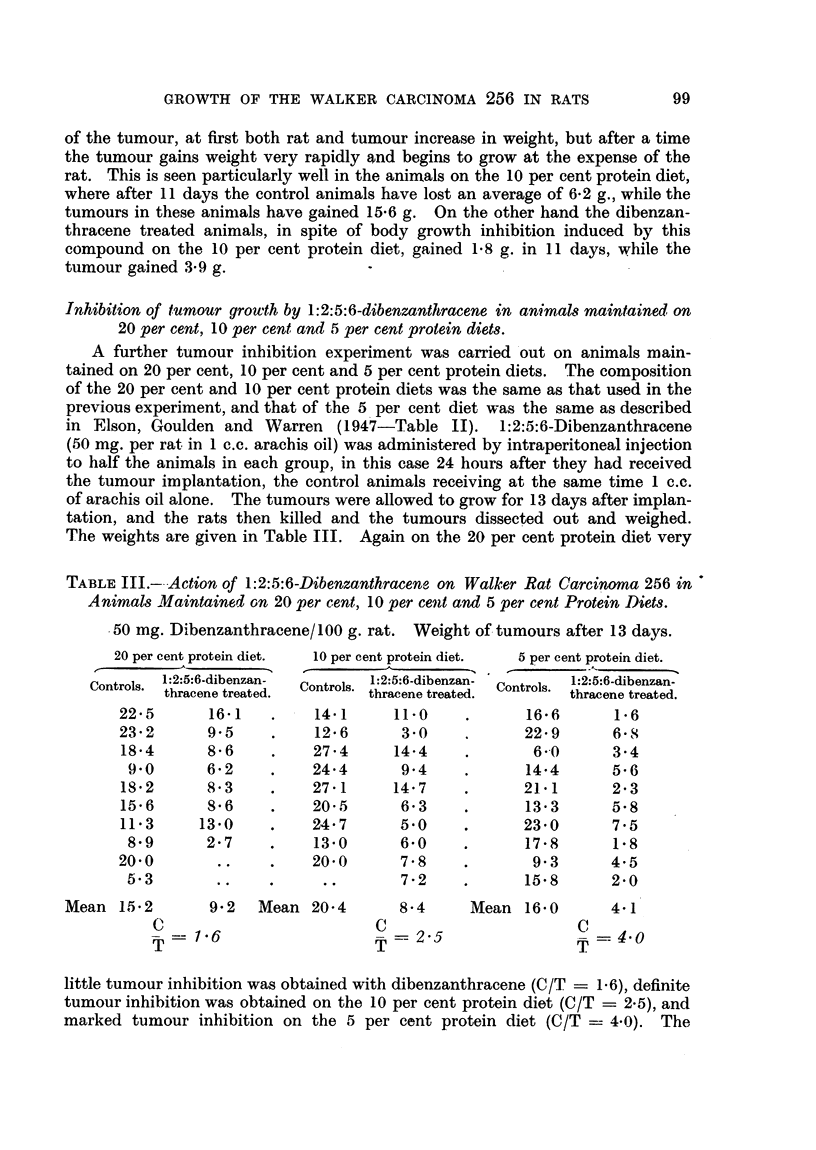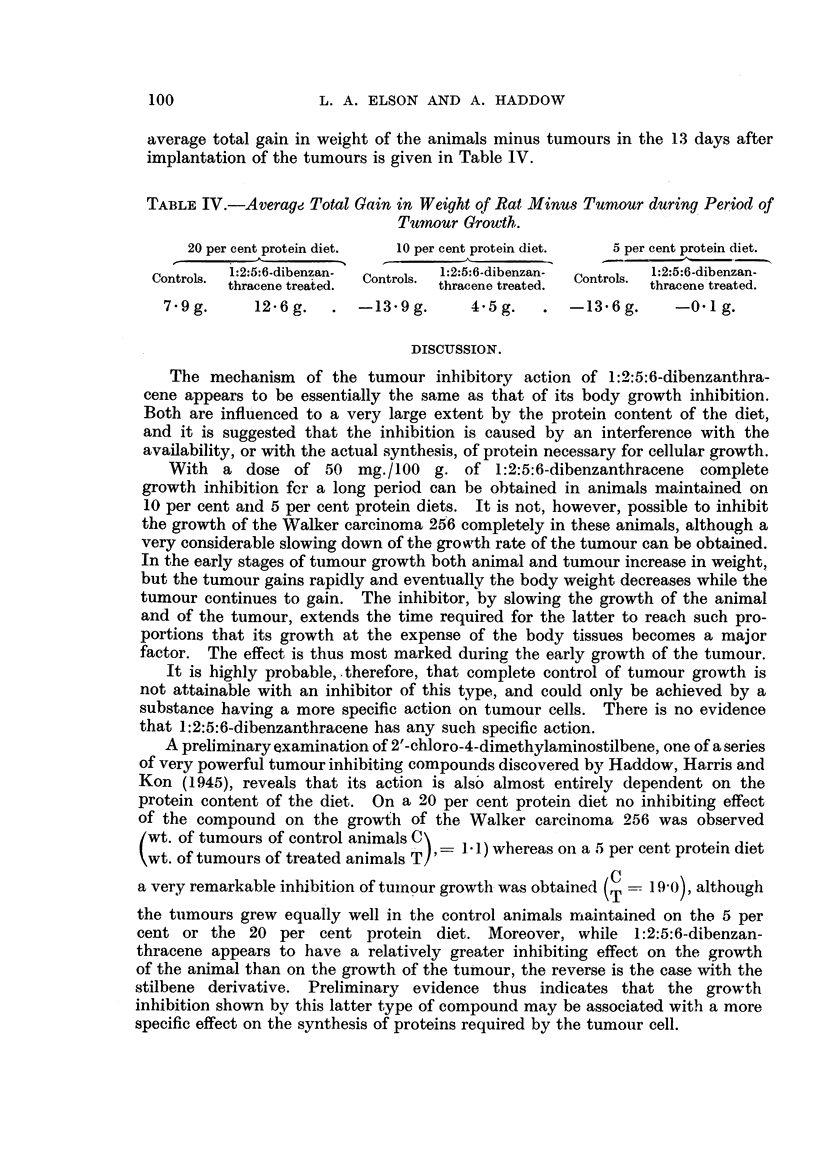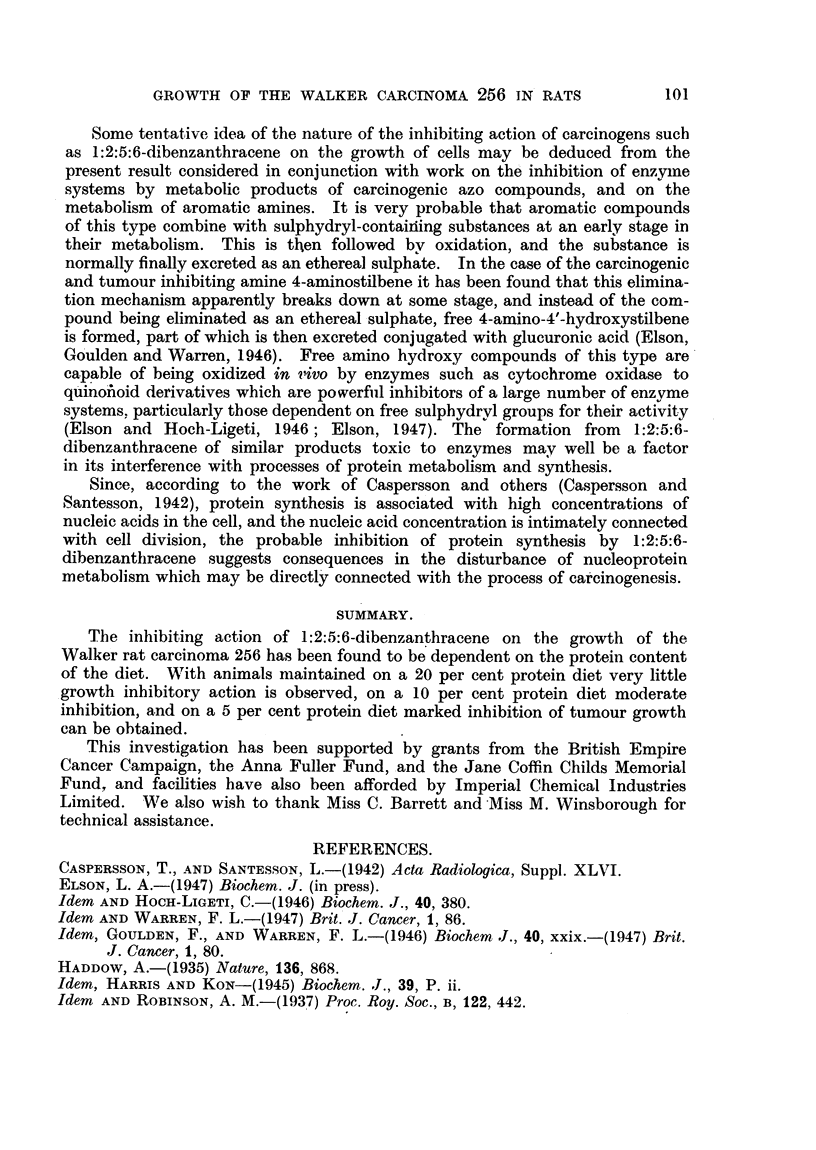# The Inhibitory Action of 1:2:5:6-Dibenzanthracene on the Growth of the Walker Carcinoma 256 in Rats Maintained on High and Low Protein Diets

**DOI:** 10.1038/bjc.1947.12

**Published:** 1947-03

**Authors:** L. A. Elson, A. Haddow


### THE INHIBITORY ACTION OF 1:2:5:6-DIBENZANTHRACENE ON THE GROWTH OF THE WALKER CARCINOMA 256 IN RATS MAINTAINED ON HIGH AND LOW PROTEIN DIETS.

L. A. ELSON AND A. HADDOW.

*From The Chester Beatty Research Institute, The Royal Cancer Hospital (Free), London, S.W. 3.*

THE protein content of the diet has been shown to have a profound effect on the inhibition of body growth of rats which is produced by 1:2:5:6-dibenzanthracene (Elson and Warren, 1947). If the tumour inhibiting properties of this hydrocarbon (Haddow, 1935; Haddow and Robinson, 1937) are related to its property of body growth inhibition, it seemed probable that the degree of tumour inhibition obtained with it might also be directly influenced by the protein content of the diet. Experiments to test this have now been carried out.

### EXPERIMENTAL.

### *Inhibition of tumour growth by* 1:2:5:6-*dibenzanthracene in animals maintained on* 20 *per cent and* 10 *per cent protein diets.*

One group of 18 male Wistar rats was maintained on a high (20 per cent) protein diet, and another similar group of 18 animals on a low (10 per cent) protein diet. The composition of the diets was the same as those used for the body growth inhibition experiments (Elson and Warren, 1947, Table I). The animals were kept in separate cages and weighed every 2 or 3 days. After being on the diet for 14 days both groups were implanted with the Walker carcinoma 256, and 9 animals of each group were later the same day also given an intraperitoneal injection of 50 mg. 1:2:5:6-dibenzanthracene in 1 c.c. arachis oil, the remaining 9 animals of each group acting as controls and receiving an injection of 1 c.c. arachis oil alone. The weighing of the animals was continued during the development of the tumours, and an index of the size of the tumours obtained after

7 days and again after 10 days by measuring along two axes with calipers. According to these measurements very little difference was observed between the size of the tumours of the control and dibenzanthracene treated animals in the high protein group after 7 days, and the tumours of the treated animals appeared to be only slightly smaller than those of the controls after 10 days.

In the low protein group, however, the measurements on the 7th and on the 10th days showed that there was considerable inhibition of tumour growth in the animals treated with dibenzanthracene, while the tumours of the control animals had grown to about the same size as those of the controls of the high protein group. After 11 days the animals were killed and the tumours dissected out and weighed. The weights are given in Table I. Very little tumour inhibition was obtained when the animals were maintained on the high protein diet, the ratio of the average weight of the tumours of the control animals to the average weight of the tumours of the dibenzanthracene treated animals (C/T) being 1·8, whereas in the animals maintained on the low protein diet a marked inhibition of tumour growth occurred (C/T = 4·0).

TABLE I.—*Action of* 1:2:5:6-*Dibenzanthracene on Walker Rat Carcinoma* 256 *in Animals Maintained on* 20 *per cent and* 10 *per cent Protein Diets.*

50 mg. Dibenzanthracene/100 g. rat. Weight of tumours after 11 days.

| High (20 per cent) protein diet. | | Low (10 per cent) protein diet. | |
|---|---|---|---|
| Controls. | 1:2:5:6-dibenzanthracene treated. | Controls. | 1:2:5:6-dibenzanthracene treated. |
| 10·5 g. | 5·5 g. | 20·5 g. | 3·0 g. |
| 16.0 g. | 13·0 g. | 10·5 g. | 5·0 g. |
| 13·5 g. | 7·5 g. | 14·0 g. | 2·0 g. |
| 17·0 g. | 7·5 g. | 17·0 g. | 6·0 g. |
| 11·0 g. | 3·0 g. | 17·0 g. | 3·0 g. |
| 14·0 g. | 11·0 g. | 13·0 g. | 4·0 g. |
| 18·5 g. | 11·0 g. | 18·0 g. | 2·0 g. |
| 19·0 g. | 5·0 g. | 14·5 g. | 4·5 g. |
| 11·0 g. | 7·0 g. | .. | 5·5 g. |
| Mean *14·5 g.* | Mean *7·9 g.* | Mean *15·6 g.* | Mean *3·9g.* |
| $\frac{C}{T} = 1·8$ | | $\frac{C}{T} = 4·0$ | |

By subtracting the weight of the tumour from the weight of the tumour-bearing rat when killed, the average total gain in weight of the animal minus tumour in the 11 days after implantation of the tumour was obtained and is given in Table II. In considering the growth of the rat in relation to the growth

TABLE II.—*Average Total Gain in Weight of Rat Minus Tumour during Period of Tumour Growth.*

| High (20 per cent) protein diet. | | Low (10 per cent) protein diet. | |
|---|---|---|---|
| Controls. | 1:2:5:6-dibenxanthracene treated. | Controls. | 1:2:5:6-dibenzanthracene treated. |
| 4·4 g. | 8·6 g. | —6·2 g. | 1·8 g. |

of the tumour, at first both rat and tumour increase in weight, but after a time the tumour gains weight very rapidly and begins to grow at the expense of the rat. This is seen particularly well in the animals on the 10 per cent protein diet, where after 11 days the control animals have lost an average of 6·2 g., while the tumours in these animals have gained 15·6 g. On the other hand the dibenzanthracene treated animals, in spite of body growth inhibition induced by this compound on the 10 per cent protein diet, gained 1·8 g. in 11 days, while the tumour gained 3·9 g.

*Inhibition of tumour growth by* 1:2:5:6-*dibenzanthracene in animals maintained on* 20 *per cent,* 10 *per cent and* 5 *per cent protein diets.*

A further tumour inhibition experiment was carried out on animals maintained on 20 per cent, 10 per cent and 5 per cent protein diets. The composition of the 20 per cent and 10 per cent protein diets was the same as that used in the previous experiment, and that of the 5 per cent diet was the same as described in Elson, Goulden and Warren (1947—Table II). 1:2:5:6-Dibenzanthracene (50 mg. per rat in 1 c.c. arachis oil) was administered by intraperitoneal injection to half the animals in each group, in this case 24 hours after they had received the tumour implantation, the control animals receiving at the same time 1 c.c. of arachis oil alone. The tumours were allowed to grow for 13 days after implantation, and the rats then killed and the tumours dissected out and weighed. The weights are given in Table III. Again on the 20 per cent protein diet very

TABLE III.—*Action of* 1:2:5:6-*Dibenzanthracene on Walker Rat Carcinoma* 256 *in Animals Maintained on* 20 *per cent,* 10 *per cent and* 5 *per cent Protein Diets.*

50 mg. Dibenzanthracene/100 g. rat. Weight of tumours after 13 days.

| | 20 per cent protein diet. | | | 10 per cent protein diet. | | | 5 per cent protein diet. | |
|---|---|---|---|---|---|---|---|---|
| | Controls. | 1:2:5:6-dibenzanthracene treated. | | Controls. | 1:2:5:6-dibenzanthracene treated. | | Controls. | 1:2:5:6-dibenzanthracene treated. |
| | 22·5 | 16·1 | | 14·1 | 11·0 | | 16·6 | 1·6 |
| | 23·2 | 9·5 | | 12·6 | 3·0 | | 22·9 | 6·8 |
| | 18·4 | 8·6 | | 27·4 | 14·4 | | 6·0 | 3·4 |
| | 9·0 | 6·2 | | 24·4 | 9·4 | | 14·4 | 5·6 |
| | 18·2 | 8·3 | | 27·1 | 14·7 | | 21·1 | 2·3 |
| | 15·6 | 8·6 | | 20·5 | 6·3 | | 13·3 | 5·8 |
| | 11·3 | 13·0 | | 24·7 | 5·0 | | 23·0 | 7·5 |
| | 8·9 | 2·7 | | 13·0 | 6·0 | | 17·8 | 1·8 |
| | 20·0 | .. | | 20·0 | 7·8 | | 9·3 | 4·5 |
| | 5·3 | .. | | .. | 7·2 | | 15·8 | 2·0 |
| Mean | 15·2 | 9·2 | Mean | 20·4 | 8·4 | Mean | 16·0 | 4·1 |
| | $\frac{C}{T} = 1{\cdot}6$ | | | $\frac{C}{T} = 2{\cdot}5$ | | | $\frac{C}{T} = 4{\cdot}0$ | |

little tumour inhibition was obtained with dibenzanthracene (C/T = 1·6), definite tumour inhibition was obtained on the 10 per cent protein diet (C/T = 2·5), and marked tumour inhibition on the 5 per cent protein diet (C/T = 4·0). The average total gain in weight of the animals minus tumours in the 13 days after implantation of the tumours is given in Table IV.

TABLE IV.—*Averagɛ Total Gain in Weight of Rat Minus Tumour during Period of Tumour Growth.*

| 20 per cent protein diet. | | 10 per cent protein diet. | | 5 per cent protein diet. | |
|---|---|---|---|---|---|
| Controls. | 1:2:5:6-dibenzanthracene treated. | Controls. | 1:2:5:6-dibenzanthracene treated. | Controls. | 1:2:5:6-dibenzanthracene treated. |
| 7·9 g. | 12·6 g. | —13·9 g. | 4·5 g. | —13·6 g. | —0·1 g. |

DISCUSSION.

The mechanism of the tumour inhibitory action of 1:2:5:6-dibenzanthracene appears to be essentially the same as that of its body growth inhibition. Both are influenced to a very large extent by the protein content of the diet, and it is suggested that the inhibition is caused by an interference with the availability, or with the actual synthesis, of protein necessary for cellular growth.

With a dose of 50 mg./100 g. of 1:2:5:6-dibenzanthracene complete growth inhibition for a long period can be obtained in animals maintained on 10 per cent and 5 per cent protein diets. It is not, however, possible to inhibit the growth of the Walker carcinoma 256 completely in these animals, although a very considerable slowing down of the growth rate of the tumour can be obtained. In the early stages of tumour growth both animal and tumour increase in weight, but the tumour gains rapidly and eventually the body weight decreases while the tumour continues to gain. The inhibitor, by slowing the growth of the animal and of the tumour, extends the time required for the latter to reach such proportions that its growth at the expense of the body tissues becomes a major factor. The effect is thus most marked during the early growth of the tumour.

It is highly probable, therefore, that complete control of tumour growth is not attainable with an inhibitor of this type, and could only be achieved by a substance having a more specific action on tumour cells. There is no evidence that 1:2:5:6-dibenzanthracene has any such specific action.

A preliminary examination of 2′-chloro-4-dimethylaminostilbene, one of a series of very powerful tumour inhibiting compounds discovered by Haddow, Harris and Kon (1945), reveals that its action is also almost entirely dependent on the protein content of the diet. On a 20 per cent protein diet no inhibiting effect of the compound on the growth of the Walker carcinoma 256 was observed $\left(\frac{\text{wt. of tumours of control animals C}}{\text{wt. of tumours of treated animals T}}, = 1\cdot1\right)$ whereas on a 5 per cent protein diet a very remarkable inhibition of tumour growth was obtained $\left(\frac{C}{T} = 19\cdot0\right)$, although the tumours grew equally well in the control animals maintained on the 5 per cent or the 20 per cent protein diet. Moreover, while 1:2:5:6-dibenzanthracene appears to have a relatively greater inhibiting effect on the growth of the animal than on the growth of the tumour, the reverse is the case with the stilbene derivative. Preliminary evidence thus indicates that the growth inhibition shown by this latter type of compound may be associated with a more specific effect on the synthesis of proteins required by the tumour cell.

Some tentative idea of the nature of the inhibiting action of carcinogens such as 1:2:5:6-dibenzanthracene on the growth of cells may be deduced from the present result considered in conjunction with work on the inhibition of enzyme systems by metabolic products of carcinogenic azo compounds, and on the metabolism of aromatic amines. It is very probable that aromatic compounds of this type combine with sulphydryl-containing substances at an early stage in their metabolism. This is then followed by oxidation, and the substance is normally finally excreted as an ethereal sulphate. In the case of the carcinogenic and tumour inhibiting amine 4-aminostilbene it has been found that this elimination mechanism apparently breaks down at some stage, and instead of the compound being eliminated as an ethereal sulphate, free 4-amino-4′-hydroxystilbene is formed, part of which is then excreted conjugated with glucuronic acid (Elson, Goulden and Warren, 1946). Free amino hydroxy compounds of this type are capable of being oxidized *in vivo* by enzymes such as cytochrome oxidase to quinonoid derivatives which are powerful inhibitors of a large number of enzyme systems, particularly those dependent on free sulphydryl groups for their activity (Elson and Hoch-Ligeti, 1946; Elson, 1947). The formation from 1:2:5:6-dibenzanthracene of similar products toxic to enzymes may well be a factor in its interference with processes of protein metabolism and synthesis.

Since, according to the work of Caspersson and others (Caspersson and Santesson, 1942), protein synthesis is associated with high concentrations of nucleic acids in the cell, and the nucleic acid concentration is intimately connected with cell division, the probable inhibition of protein synthesis by 1:2:5:6-dibenzanthracene suggests consequences in the disturbance of nucleoprotein metabolism which may be directly connected with the process of carcinogenesis.

### SUMMARY.

The inhibiting action of 1:2:5:6-dibenzanthracene on the growth of the Walker rat carcinoma 256 has been found to be dependent on the protein content of the diet. With animals maintained on a 20 per cent protein diet very little growth inhibitory action is observed, on a 10 per cent protein diet moderate inhibition, and on a 5 per cent protein diet marked inhibition of tumour growth can be obtained.

This investigation has been supported by grants from the British Empire Cancer Campaign, the Anna Fuller Fund, and the Jane Coffin Childs Memorial Fund, and facilities have also been afforded by Imperial Chemical Industries Limited. We also wish to thank Miss C. Barrett and Miss M. Winsborough for technical assistance.

### REFERENCES.

Caspersson, T., and Santesson, L.—(1942) *Acta Radiologica*, Suppl. XLVI.
Elson, L. A.—(1947) *Biochem. J.* (in press).
*Idem* and Hoch-Ligeti, C.—(1946) *Biochem. J.*, **40**, 380.
*Idem* and Warren, F. L.—(1947) *Brit. J. Cancer*, **1**, 86.
*Idem*, Goulden, F., and Warren, F. L.—(1946) *Biochem J.*, **40**, xxix.—(1947) *Brit. J. Cancer*, **1**, 80.
Haddow, A.—(1935) *Nature*, **136**, 868.
*Idem*, Harris and Kon—(1945) *Biochem. J.*, **39**, P. ii.
*Idem* and Robinson, A. M.—(1937) *Proc. Roy. Soc.*, B, **122**, 442.